# Real-Time Reverse Transcription Recombinase-Aided Amplification Assay for Rapid Amplification of the *N* Gene of SARS-CoV-2

**DOI:** 10.3390/ijms232315269

**Published:** 2022-12-03

**Authors:** Huan Cui, Fei Tu, Cheng Zhang, Chunmao Zhang, Kui Zhao, Juxiang Liu, Shishan Dong, Ligong Chen, Jun Liu, Zhendong Guo

**Affiliations:** 1 Changchun Veterinary Research Institute, Chinese Academy of Agriculture Sciences, Changchun 130122, China; 2 College of Animal Medicine, Jilin University, Changchun 130062, China; 3 College of Veterinary Medicine, Hebei Agricultural University, Baoding 071000, China

**Keywords:** reverse transcription recombinase-aided amplification (RT-RAA), SARS-CoV-2, isothermal amplification, rapid diagnosis, visual amplification

## Abstract

COVID-19 was officially declared a global pandemic disease on 11 March 2020, with severe implications for healthcare systems, economic activity, and human life worldwide. Fast and sensitive amplification of the severe acute respiratory syndrome virus 2 (SARS-CoV-2) nucleic acids is critical for controlling the spread of this disease. Here, a real-time reverse transcription recombinase-aided amplification (RT-RAA) assay, targeting conserved positions in the nucleocapsid protein gene (*N* gene) of SARS-CoV-2, was successfully established for SARS-CoV-2. The assay was specific to SARS-CoV-2, and there was no cross-reaction with other important viruses. The sensitivity of the real-time RT-RAA assay was 142 copies per reaction at 95% probability. Furthermore, 100% concordance between the real-time RT-RAA and RT-qPCR assays was achieved after testing 72 clinical specimens. Further linear regression analysis indicated a significant correlation between the real-time RT-RAA and RT-qPCR assays with an R2 value of 0.8149 (*p* < 0.0001). In addition, the amplicons of the real-time RT-RAA assay could be directly visualized by a portable blue light instrument, making it suitable for the rapid amplification of SARS-CoV-2 in resource-limited settings. Therefore, the real-time RT-RAA method allows the specific, sensitive, simple, rapid, and reliable detection of SARS-CoV-2.

## 1. Introduction

Severe acute respiratory syndrome virus 2 (SARS-CoV-2) has severely affected global health and the world economy for two and a half years. Coronaviruses belong to the family Coronaviridae and the genus Coronaviridae and are divided into four genera, namely α, β, γ, and δ [1]. SARS-CoV-2 is a β-coronavirus, and it is the seventh type of coronavirus known to infect human beings, in addition to HCoV-229E, HCoV-NL63, HCoV-HKU1, HCoV-OC43, SARS-CoV, and MERS-CoV [2,3]. Coronaviruses, the largest of all RNA viruses, are positive-sense single-stranded RNA (+ssRNA) viruses with a genome length of approximately 27–32 kb [2]. The genome of SARS-CoV-2 is approximately 29.9 kb long and contains 4 structural proteins (S, E, M, and N) and 16 nonstructural proteins (nsp1–16) [4,5]. SARS-CoV-2 is highly contagious and can be transmitted from person to person via the aerosol route, which has accelerated its global spread and increased the difficulty of prevention and control [6,7,8]. Globally, as of 19 August 2022, there have been 591,683,619 confirmed cases of COVID-19, including 6,443,306 deaths, reported to the World Health Organization (WHO). Rapid and sensitive diagnosis of SARS-CoV-2 is critical for controlling the spread of this disease. RT-qPCR is a sensitive tool for detecting SARS-CoV-2 [9]. However, the complex operation and expensive equipment requirements limit its application in resource-limited environments. Thus, it is necessary to develop a portable, simple, low-cost, and sensitive on-site diagnostic method to detect SARS-CoV-2.

In vitro nucleic acid amplification is the basis of molecular biological research, and PCR, qPCR, and other technologies are the most widely used methods, but these methods all require complex thermal cyclers, which limits their application when medical resources are scarce and in emergency situations. Nucleic acid isothermal amplification technology has potential application prospects under medical resource scarcity and for on-site rapid amplification because this amplification reaction can be carried out without a complicated thermal cycler. Recombinase-aided amplification (RAA), a new isothermal amplification technique, can be completed within 30 min at 37–42 °C. There are three key enzymes in the RAA system: a single-stranded DNA-binding protein (SSB), which is a protein specifically responsible for binding to single-stranded regions of DNA; a recombinase, which pairs the specific primers with template DNA; and a strand-displacing DNA polymerase for extension and DNA amplification [10,11]. In the RAA system, the addition of specific fluorescent probes can be used for real-time monitoring of DNA amplicons. Furthermore, adding reverse transcriptase and specific fluorescent probes to the RAA system allows the real-time monitoring of RNA amplicons [12,13,14]. The CRISPR system can be combined with RAA technology to achieve ultrahigh sensitivity and specific detection of DNA or RNA molecules [15,16,17]. Due to its simple primer design, fast amplification speed, high sensitivity, low equipment requirements, lack of a need for expensive instruments, simple operation, and visual output of results, RAA has been widely used to detect various pathogenic microorganisms [13,18,19].

Here, a real-time RT-RAA assay was successfully applied for the detection of SARS-CoV-2. This assay is highly sensitive, specific, and easy to operate. The amplicons can be detected by the naked eye with a portable blue-light instrument (an excitation wavelength of 480 nm), making the assay suitable for amplifying the *N* gene of SARS-CoV-2 under medical resource scarcity and in emergency situations. Furthermore, we compared the amplification performance of the real-time RT-RAA and RT-qPCR methods using clinical samples.

## 2. Results

### 2.1. Positions of the Real-Time RT-RAA Primers and Probe

**Figure 1:**
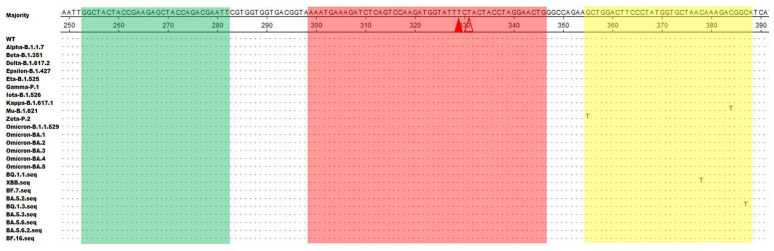
Positions of the real-time RT-RAA primers and probe in the nucleocapsid protein gene (*N* gene) sequences of different SARS-CoV-2 strains in the GenBank database. Dots represent nucleotide residues that match the majority. The forward primer (F253–282) is shaded in green, the reverse primer (R355–388) is in yellow, and the exo probe (p299–346) is in red. The two T residues within p299–346 labeled with a fluorophore (FAM) and quencher (BHQ1) are marked with solid and hollow triangles, respectively.

The two modified thymine (T) residues in our chosen probe (p299–346) were fully conserved among the twenty-six representative SARS-CoV-2 strains (Figure 1). After secondary primer screening, the optimal primer pair F253–282/R355–388 was screened out. In the figure, dots indicate nucleotide residues that matched the majority, the forward primer (F253–282) is shaded in green, the reverse primer (R355–388) is in yellow, and the exo probe (p299–346) is in red. The two T residues within p299–346 labeled with a fluorophore (FAM) and quencher (BHQ1) are labeled with solid and hollow triangles, respectively (Figure 1).

### 2.2. Screening the Optimal Primers for Real-Time RT-RAA Amplification

**Figure 2:**
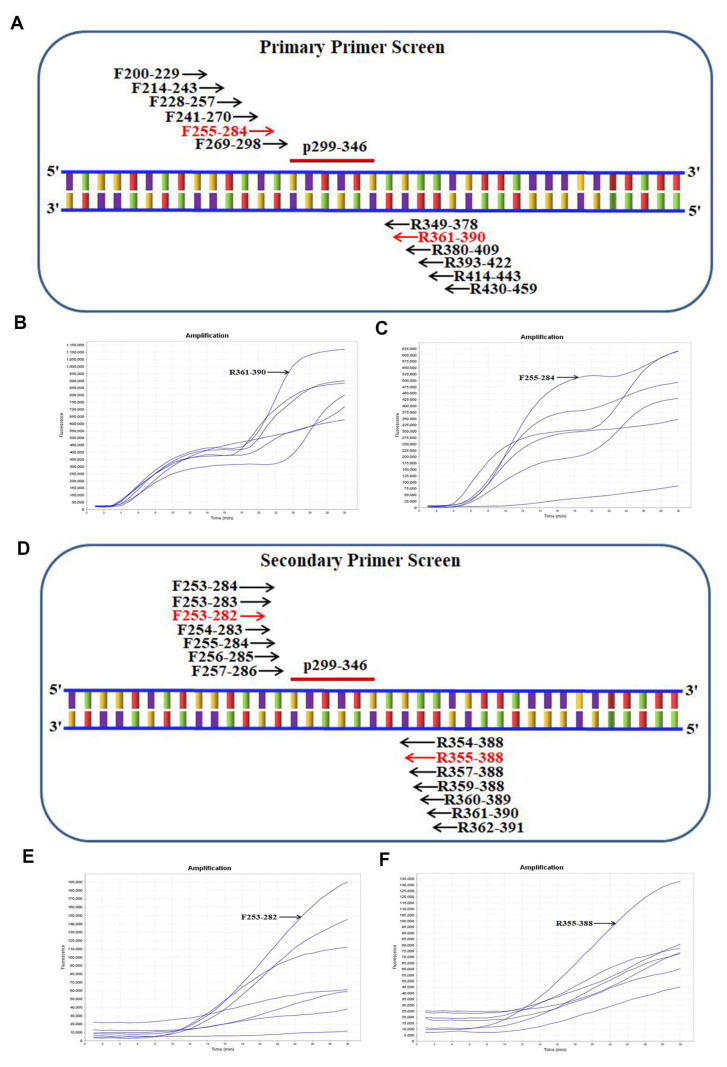
Screening the optical primers for real-time RT-RAA detection. (**A**) Sketch map of primary primer screening. In the primer name, the numbers indicate the position within the *N* gene from hCoV-19/Wuhan/WIV04/2019 (EPI_ISL_402,124). (**B**) Primary reverse primer screening results. The forward primer F200–229 was randomly selected to screen all six reverse primers. (**C**) Real-time RT-RAA primary forward primer screening results. The selected reverse primer R361–390 was used to screen all six forward primers. (**D**) Sketch map of secondary primer screening. (**E**) Secondary forward primer screening results. The picked reverse primer R361–390 was applied to screen all seven forward primers. (**F**) Secondary reverse primer screening results. The selected forward primer F253–282 was used to screen all seven reverse primers.

After primary primer screening, the optimal primer pair F255–284/R361–390 was screened out (Figure 2A–C). After secondary primer screening, the optimal primer pair F253–282/R355–388 was screened out (Figure 2D–F).

### 2.3. Specificity Analysis

**Figure 3:**
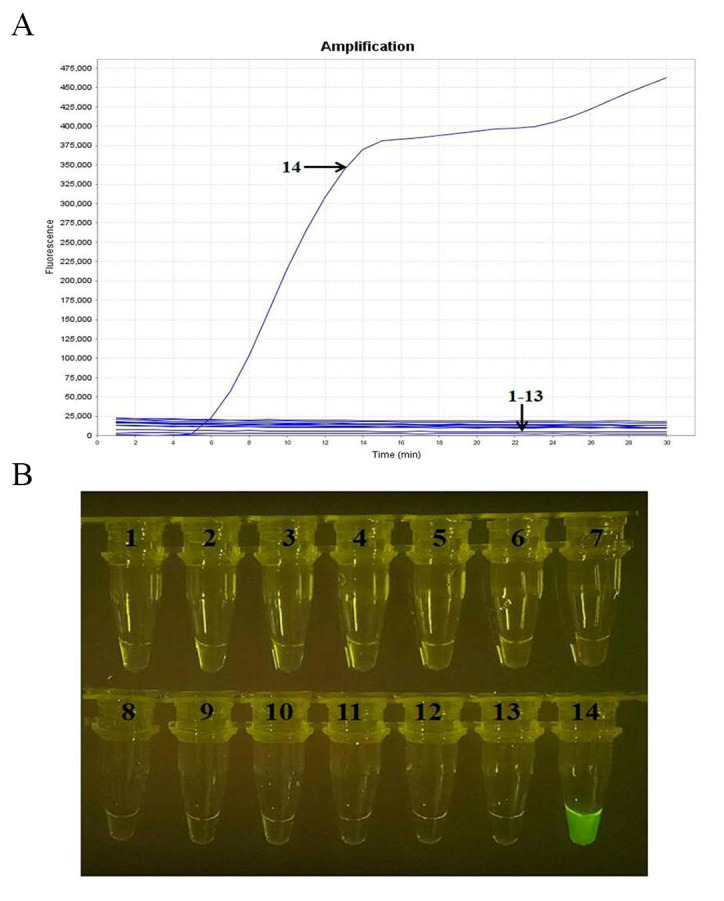
Specificity tests for SARS-CoV-2. (**A**) Real-time RT-RAA amplification results via real-time fluorescence read-out. (**B**) The amplicons of the real-time RT-RAA assay were detected by the naked eye with a portable blue-light instrument. Curves or tubes 1–14, nucleic acid templates corresponding to H1N1-CA04, H1N1-SC99, H3N2, H9N2, IBV-Y, IBV-V, RSV-A, RSV-B, HCoV-229E, HCoV-OC43, HCoV-NL63, and HCoV-HKU1, negative control, and SARS-CoV-2, respectively.

Specificity analysis showed that the real-time RT-RAA assay was able to detect SARS-CoV-2 but not H1N1-CA04, H1N1-SC99, H3N2, IBV-Y, IBV-V, H9N2, RSV-A, RSV-B, HCoV-229E, HCoV-OC43, HCoV-NL63, and HCoV-HKU1, nor the negative control (Figure 3A). In addition, the amplified products could be visualized by a TGreen instrument (Figure 3B). These results indicate that the established real-time RT-RAA assay is specific to SARS-CoV-2.

### 2.4. Sensitivity Analysis

**Figure 4:**
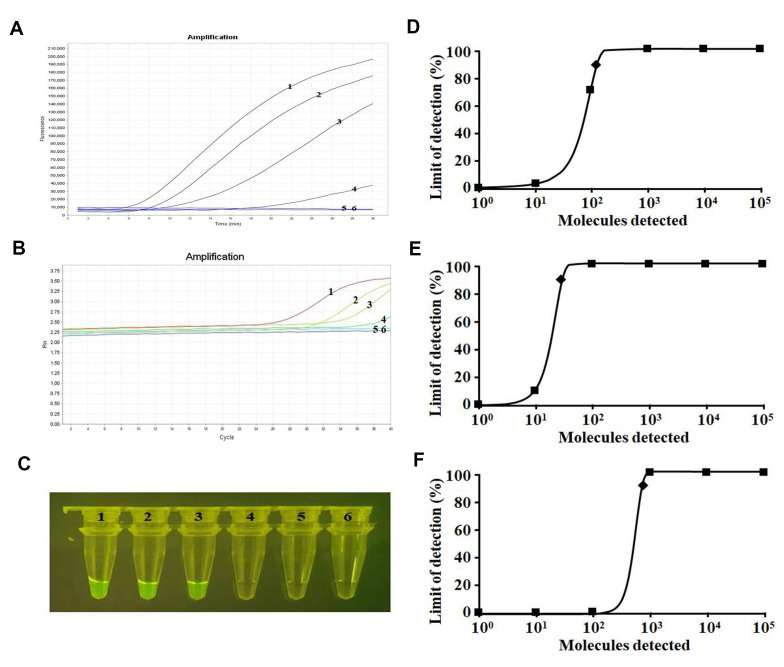
Sensitivity tests for SARS-CoV-2. Curve/Tubes 1–6, corresponding to 10^5^–10^1^ copies and the negative control, respectively. (**A**) Results of real-time RT-RAA amplification by real-time fluorescence read-out. (**B**) Results of the RT-qPCR assay. (**C**) Results of the real-time RT-RAA assay by visualization. (**D**) The detection limit of the real-time RT-RAA assay at 95% reliability (142 copies per reaction) is labeled with a rhomboid. (**E**) The detection limit of the RT-qPCR assay at 95% reliability (38 copies per reaction) is labeled with a rhomboid. (**F**) The detection limit of the real-time RT-RAA combined with visualization at 95% reliability (794 copies per reaction) is labeled with a rhomboid.

Sensitivity analysis showed that the amplification limit was 100 copies per reaction by means of a real-time fluorescence read-out in both the real-time RT-RAA assay and RT-qPCR assay (Figure 4A,B). The visualized results of the real-time RT-RAA assay showed a sensitivity of 1000 copies per reaction (Figure 4C). Probit regression analyses further indicated that the amplification limits of the real-time RT-RAA and RT-qPCR assays and visual judgment sensitivity of the real-time RT-RAA assay at 95% probability were 142, 38, and 794 copies per reaction for SARS-CoV-2, respectively (Figure 4D–F).

### 2.5. Amplification of Clinical Samples

**Figure 5:**
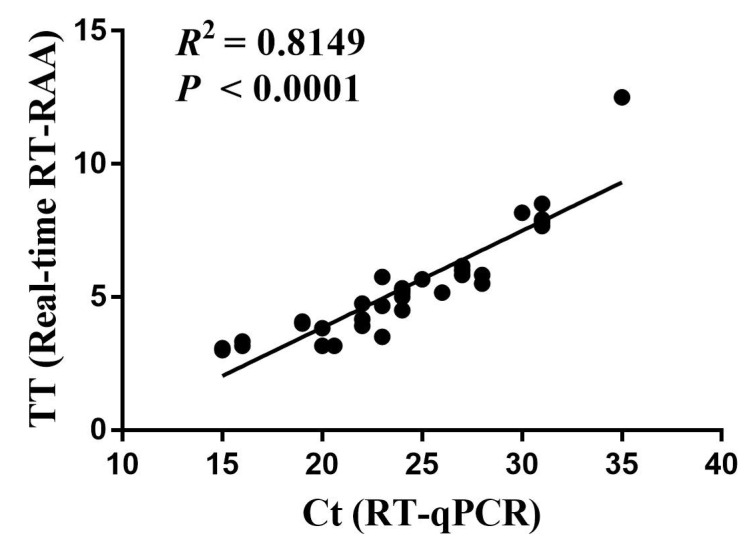
Comparison between real-time RT-RAA and RT-qPCR for the detection of SARS-CoV-2 in clinical samples. Thirty-six SARS-CoV-2 positive samples were used to evaluate the consistency between the two methods. The real-time RT-RAA threshold time (TT) values (*y* axis) and the RT-qPCR cycle threshold (Ct) values (*x* axis) were analyzed by linear regression with GraphPad Prism software. The *R*^2^ value was 0.8149, *p* < 0.0001.

Real-time RT-RAA and RT-qPCR were performed on 72 clinical oropharyngeal swab samples to assess clinical performance. Compared with RT-qPCR, the sensitivity and specificity of the real-time RT-RAA (via real-time fluorescence read-out) assay were 100% (36/36) and 100.0% (36/36), respectively. The two assays showed a very good correlation, with a Kappa value of 1 (*p* < 0.001, Table 1). The positive predictive value (PPV) and the negative predictive value (NPV) were both 100%. Furthermore, the sensitivity and specificity of the real-time RT-RAA (via visual detection) assay were 97.22% (35/36) and 100.0% (36/36), respectively. The two assays showed a very good correlation, with a Kappa value of 0.972 (*p* < 0.001, Table 1). The PPV was 100.0%, while the NPV was 97%. Further linear regression analysis indicated that a significant correlation exists between the real-time RT-RAA and RT-qPCR assays, with an *R*^2^ value of 0.8149 (*p* < 0.0001, Figure 5). This demonstrates that the developed real-time RT-RAA assay can rapidly amplify the *N* gene of SARS-CoV-2 in resource-limited settings.

## 3. Discussion

The COVID-19 pandemic has had serious implications for the global economy, public health, and human life [20]. China has effectively controlled the spread of this disease to date. However, SARS-CoV-2 remains an active epidemic virus in many countries around the world [21,22]. Fast and reliable detection of SARS-CoV-2 is critical for controlling the spread of this disease. RT-qPCR amplification is currently the main tool for the detection of SARS-CoV-2 in China and other countries [9], and it plays an important role in controlling and preventing the spread of SARS-CoV-2. Despite the high specificity and sensitivity of the RT-qPCR test, false negative results in symptomatic patients and/or patients with positive CT scans remain a challenge [23]. In addition, it requires complex and expensive devices and trained personnel and is time consuming. These disadvantages make it unsuitable for widespread use in the field. Therefore, it is necessary to develop a faster, portable, and reliable on-site diagnostic method for the amplification of SARS-CoV-2.

The mutation and recombination of the virus genome have brought great difficulties to the detection of SARS-CoV-2. The five regions of the SARS-CoV-2 genome are widely used to design primers, including nucleocapsid (N), RNA polymerase-dependent RNA (RdRp), ORF1ab, envelope (E), and spike (S). Among them, ORF1ab, N, and RdRp primers have high sensitivity, specificity, and positive predictive value [24]. Here, we developed a novel real-time RT-RAA assay targeting the *N* gene (highly conserved) for the rapid detection of SARS-CoV-2. We adopted the primer screening strategy reported in previous studies [11]. Briefly, we used a forward primer (randomly selected) to screen all of the reverse primers, and then the best reverse primer was selected and used to screen all of the forward primers. After secondary primer screening, the optimal primer combination F253–282/R355–388 was screened out. The real-time RT-RAA assay exhibited an amplification limit of 142 copies of recombinant plasmid per reaction at 95% probability, which is similar to that of the RT-qPCR assay developed by the China CDC (amplification limit of 38 copies of recombinant plasmid per reaction at 95% probability). Among 72 clinical tissue samples, 36 and 36 samples tested positive for SARS-CoV-2 upon real-time RT-RAA and RT-qPCR, respectively. The overall agreement between the two assays was 100% (36/36). One sample tested negative using the real-time RT-RAA (via visual detection) assay but positive in the real-time RT-RAA (via real-time fluorescence read-out) and RT-qPCR assays, because this sample’s Ct value was 35.

Because the RAA system contains reverse transcriptase, the reverse transcription reaction and RAA amplification are carried out simultaneously, and the direct amplification of RNA molecules can be completed within 30 min at 42 °C. On the other hand, RT-qPCR requires at least 1.5 h to complete the amplification. Interestingly, the amplicon of this real-time RT-RAA assay can be visually detected with a portable blue-light instrument, making it suitable for SARS-CoV-2 amplification under medical resource constraints and in emergency situations. In addition, we found that the reaction volume for real-time RT-RAA assays could be reduced from 50 μL (total volume recommended by the manufacturer’s instructions) to 25 μL with no effect on the assay results for clinical samples. This suggests that the cost of real-time RT-RAA amplification can be further reduced by reducing the reaction volume. In addition, real-time RT-RAA amplification can also be combined with microfluidic chip technology for multipathogen detection [25]. In addition, several studies have shown that the combination of RAA technology and the CRISPR system can achieve ultrahigh sensitivity and specific detection of single DNA or RNA molecules [26,27,28]. Because RAA technology is specific, sensitive, rapid, and easy to operate, it has great application prospects in the early diagnosis of animal diseases, immediate detection, and import- and export-related quarantine.

## 4. Materials and Methods

### 4.1. Virus and Clinical Samples of SARS-CoV-2

SARS-CoV-2 BetaCoV/Beijing/IME-BJ05–2020 (Biological Sample Library: SAMC138020) and seventy-two clinical samples (36 RT-qPCR positive samples and 36 RT-qPCR negative samples collected from Wuhan Huoshenshan Hospital in 2020, oropharyngeal swab) were stored in the biosafety level 3 laboratory of Changchun Veterinary Research Institute, Chinese Academy of Agricultural Sciences. All samples were processed in the biosafety level 3 laboratory.

### 4.2. Primers and Probe Design for Real-Time RT-RAA

The *N* gene sequences of 26 different strains of SARS-CoV-2 (from the GenBank database) were aligned using DNASTAR software. SnapGene software (Version 4.3.6) was used to design primers and probes. The design of RAA primers followed the following basic principles: the primer length should be greater than or equal to 30 bp, preferably between 30 and 38 bp; the length of the amplicon should not exceed 500 bp, preferably between 100 and 200 bp; the GC content should be greater than 30% and less than 70%, preferably between 40% and 60%; the base at the 3’ end of the primer should be highly conserved; it is best to avoid short sequences with many repeats in the primer; and primer sequences that directly form a hairpin structure or dimers should be avoided. The optimal primers and probe were screened according to a previously reported method [11]. Furthermore, the primers and probe for the *N*-based RT-qPCR assay of SARS-CoV-2 were also synthesized, as reported by the China CDC (Table 2). All primers and probes were synthesized by Comate Biotech Co., Ltd. (Changchun, China). Details of the final primers and probes for real-time RT-RAA amplification that we designed in this study are shown in Figure 1 and Table 2.

### 4.3. Screening the Optimal Primers for Real-Time RT-RAA Amplification

First, we picked an ideal probe (p299–346) (Figure 2, Table 2). Then, six forward (F200–229, F214–243, F228–257, F241–270, F255–284, F269–298) and six reverse (R349–378, R361–390, R380–409, R393–422, R414–443, R430–459) candidate primers were designed around p299–346 (Figure 2A). The primer screening strategy was as reported in previous studies [11]. Briefly, we used the forward primer (randomly selected) to screen all six reverse primers, picking the best reverse primer and then using it to screen all of the forward primers, and a good primer pair was found. In order to obtain more sensitive primer combinations, multiple rounds of primer screening were sometimes required.

### 4.4. Nucleic Acid Extraction

Viral RNAs of SARS-CoV-2, influenza A viruses (A/California/04/2009 (H1N1), A/Sichuan/SC99/2019 (H1N1), A/Hebei/BD79/2018 (H3N2)), influenza B viruses (Victoria and Yamagata), respiratory syncytial viruses (A and B), HCoV-229E, HCoV-OC43, HCoV-NL63, HCoV-HKU1, and total RNA from each sample were extracted using TRIzol reagent (Magen, Guangzhou, China) following the manufacturer’s instructions. The extracted RNA was eluted into 50 μL of nuclease-free water and stored at −80°C until needed.

### 4.5. Real-Time RT-RAA Protocol

The assays were performed using a kit (#WLRE8208KIT) from Amp-Future Biotech Co., Ltd. (China). Briefly, the real-time RT-RAA system (25 μL per reaction) contained the following: buffer A, 14.7 μL; forward primer (10 μM), 1.0 μL; reverse primer (10 μM), 1.0 μL; exo probe (10 μM), 0.3 μL; nuclease-free water, 4.75 μL; nucleic acid template, 2.0 μL; and buffer B, 1.25 μL. The reaction tubes were placed into a 7500 Real-Time PCR System (Applied Biosystems) at 42 °C for 30 min (1 cycle per min) for real-time monitoring of fluorescence signals. Furthermore, a portable blue-light instrument with an excitation wavelength of 480 nm (TGreen, Tiangen Biotech Co., Ltd., Beijing, China) was used to visualize the amplification products.

### 4.6. RT-qPCR Assay

The 25 μL RT-qPCR system for SARS-CoV-2 contained the following: 2 × One Step PrimeScript III RT-qPCR Mix, 12.5 μL; forward primer (10 μM), 0.5 μL; reverse primer (10 μM), 0.5 μL; probe (10 μM), 0.5 μL; nuclease-free water, 9 μL; and nucleic acid template, 2.0 μL. The reaction tubes were placed into a 7500 Real-Time PCR System (Applied Biosystems) with the following settings: initial step at 52 °C for 5 min, followed by 95 °C for 10 s and 40 cycles of 95 °C for 5 s and 60 °C for 30 s.

### 4.7. Analytical Specificity

The specificity of the real-time RT-RAA assay for SARS-CoV-2 detection was evaluated by using other important pathogens, including H1N1-CA04, H1N1-SC99, H3N2, IBV-Y, IBV-V, H9N2, RSV-A, RSV-B, HCoV-229E, HCoV-OC43, HCoV-NL63, and HCoV-HKU1. The viral RNAs for HCoV-229E, HCoV-OC43, HCoV-NL63, and HCoV-HKU1 were provided by Hebei Houqi Biology Co., LTD (Baoding, China).

### 4.8. Analytical Sensitivity

After diluting the SARS-CoV-2-N plasmid (pMD18-T-N, stored in the biosafety level 3 laboratory of Changchun Veterinary Research Institute, Chinese Academy of Agricultural Sciences) 10-fold, SARS-CoV-2-N plasmid concentrations ranging from 1 × 10^5^ to 1 × 10^1^ copies per 2 μL were obtained. Two microliters of each dilution was used as a template to evaluate the real-time RT-RAA sensitivity. For comparison, the same template was tested in parallel using the RT-qPCR assay for SARS-CoV-2. For more accurate analysis of the limit of amplification, eight independent runs were performed in both assays using the dilution series (10^5^–10^1^ copies per reaction) as templates, and probit regression analysis was performed on the data using IBM’s Statistical Product and Service Solutions (SPSS) software. The copy number of *N* gene was calculated according to a previous study [29].

### 4.9. Amplification of Clinical Samples

Forty clinical oropharyngeal swab samples were tested by the real-time RT-RAA assay for SARS-CoV-2. For comparison, the same samples were tested in parallel using the RT-qPCR assay for SARS-CoV-2. The number of viral genomic RNA copies in each sample was estimated from the measured cycle threshold (Ct) based on an established standard curve [30]. The standard curve was fitted using a series of 10-fold dilutions of a standard plasmid of the SARS-CoV-2-N (pMD18-T-N, stored in the biosafety level 3 laboratory of Changchun Veterinary Research Institute, Chinese Academy of Agricultural Sciences). The fitted standard curve equation was Ct = −3.44X0 + 41.02, where X0 is the initial viral genomic RNA copy number in the reaction system.

### 4.10. Statistical Analysis

Probit regression analysis was performed at the 95% probability level using IBM’s SPSS software to determine the amplification limits. Kappa statistics were applied to compare the coincidence rates between the real-time RT-RAA and RT-qPCR assays. Statistical analyses and data plotting were performed using the GraphPad Prism software (version 8.0.2; La Jolla, CA, USA).

## 5. Conclusions

In conclusion, a real-time reverse transcription recombinase-aided amplification assay targeting the *N* gene was successfully established to detect SARS-CoV-2. This assay can be used to diagnose SARS-CoV-2 rapidly, simply, and reliably. It could serve as a powerful and valuable tool for the detection of SARS-CoV-2, especially under medical resource constraints and in emergency situations.

## Figures and Tables

**Table 1:** Comparison of the SARS-CoV-2 real-time RT-RAA with the RT-qPCR assay on clinical samples.

Assay		RT-qPCR		Sensitivity	Specificity	Kappa
		Positive	Negative			
Real-time RT-RAA (via real-time fluorescence read-out)	Positive	36	0	100%	100%	1
	Negative	0	36			
	Total (72)	36	36			
Real-time RT-RAA (via visual detection)	Positive	35	0	97.22%	100%	0.972
	Negative	1	36			
	Total (72)	36	36			

**Table 2:** The primers and probes used in SARS-CoV-2 real-time RT-RAA and RT-qPCR assays.

Primers/Probes	Sequences (5′→3′)	Gene	Position ^1^	Source
F253–282	GGCTACTACCGAAGAGCTACCAGACGAATT	N	253–282	This study
R355–388	TGCCGTCTTTGTTAGCACCATAGGGAAGTCCAGC	N	355–388	This study
p299–346 ^2^	AAATGAAAGATCTCAGTCCAAGATGGTATT(FAM-dT)(THF)(BHQ1-dT)ACTACCTAGGAACTG[C3-spacer]	N	299–346	This study
F	GGGGAACTTCTCCTGCTAGAAT	N	608–629	China CDC
R	CAGACATTTTGCTCTCAAGCTG	N	685–706	China CDC
Probe ^3^	FAM-TTGCTGCTGCTTGACAGATT-TAMRA	N	661–680	China CDC

^1^ The location of primers/probes refers to the nucleocapsid protein gene (*N* gene) from hCoV-19/Wuhan/WIV04/2019 (EPI_ISL_402,124). ^2^ FAM-dT, thymidine nucleotide carrying fluorescein; BHQ1-dT, thymidine nucleotide carrying black hole quencher 1; THF: tetrahydrofuran spacer; C3-spacer, C3-spacer at the 3′ end to block elongation. ^3^ FAM, 6-carboxyfluorescein; TAMRA, carboxytetramethylrhodamine a quenching group.

## Data Availability

The data are available on request.
